# Being female with vitiligo disease in traditional societies within North Africa

**DOI:** 10.1186/s13030-024-00299-4

**Published:** 2024-01-10

**Authors:** Mohamed Faraj Saleh Raheel, Yaser Snoubar, Wafa Saleh Mosbah

**Affiliations:** 1 Department of Social Sciences, College of Arts and Sciences, Qatar University, Doha, 2713 Qatar; 2 Faculty of Medicine, Benghazi University, Benghazi, Libya

**Keywords:** Inflammatory skin diseases, Vitiligo, Quality of life, Gender, Traditional societies

## Abstract

This study aims to assess the influence of vitiligo illness on the quality of life of female individuals residing in Ajdabiya, Libya. Through this investigation, we aim to enhance our comprehension of the potential impact of cultural norms and conventional gender roles on managing and caring for skin disorders within a North African society. Over a 20-week period, 65 female participants diagnosed with vitiligo were recruited. The participants’ quality of life was assessed using the Skindex-16 scale. This validated tool measures the impact of skin disease on several aspects of an individual’s life, including physical, emotional, and social well-being. The findings of this study indicate that the quality of life of women with vitiligo significantly differed with age, social and functional status, and economic status. However, the illness profoundly impacted patients’ lives emotionally, with clear consequences, including diminished emotional satisfaction and reduced social participation. The results of this study highlight the negative effects that vitiligo disease can have on women’s quality of life within traditional Libyan society. This investigation also indicates that cultural norms and traditional gender patterns may contribute to these effects.

## Introduction

A common depigmenting skin condition called vitiligo is thought to affect 0.5–2% of people worldwide. Melanocytes are selectively lost during the onset of the disease, resulting in the typical non-scaly, chalky-white macules. Our understanding of the pathogenesis of vitiligo, which is now categorically recognized as an autoimmune disease, has made significant strides in recent years. Although vitiligo is frequently disregarded as a cosmetic issue, it can have devastating psychological effects and significantly interfere with daily life [1, 2]. Segmental vitiligo was categorized separately from all other forms of vitiligo in 2011, and the term vitiligo was defined to refer to all varieties of nonsegmental vitiligo [1]. Patients with vitiligo experience a significant decline in their quality of life (QOL), which is exacerbated in depressed patients. Vitiligo is a depigmenting dermatosis that may in some cases attract unwanted attention to the patient [3], especially when it affects visible body parts. Despite the benign nature of vitiligo, patients have been known to attempt various treatments for their condition, acknowledging that it greatly affects their QOL. Vitiligo can give rise to significant psychosocial consequences, with anxiety emerging as a particularly serious concern. Patients with vitiligo frequently express high comorbid anxiety, with a female predominance [4]. Skin conditions are widespread and can affect a person’s QOL and wellbeing. The prevalence of skin conditions is heightened in rural regions due to the potential lack of access to fundamental healthcare services and interventions. Rural women with vitiligo are especially susceptible to solar damage due to the limited availability of protective apparel, sunscreen, and being exposed to the elements, which can increase their vulnerability [5].

Due to their chronic nature and low mortality, skin conditions are frequently undervalued despite their high frequency. Since self-image is a crucial component of personality structure and may affect mental wellbeing, dermatologist-related consultations account for a quarter of primary healthcare inquiries, reflecting the substantial physical and psychological impairments associated with these conditions. A positive self-image fosters positive emotions, while an unfavorable one can lead to feelings of anxiety, fear, anger, depression, and social maladjustment [6, 7]. Furthermore, it has been noted that self-image is predictive of overall satisfaction and, therefore, has a major impact on an individual’s QOL. In the field of dermatology, there has been a recent notable increase in the aspirations for an elevated QOL among both men and women. This is articulated by a heightened desire for an enhanced perception of skin quality and an improvement in the outward image that individuals project on to others. Furthermore, skin conditions may affect other important factors, such as opportunities in finding partners, the ability to perform specific tasks, and professional capabilities. Acne vulgaris, vitiligo, hair loss, as well as conditions such as psoriasis or atopic dermatitis that can affect the entire body, are among skin conditions that have been known to most negatively impact QOL and self-image, including those that are related to the visible areas of the skin [8, 9]. For women, dealing with skin disease(s), this can be a daily struggle. These conditions’ physical and psychological effects may impact their QOL [10]. Dermatological afflictions may pose not only a source of annoyance for women but also a potential detriment to self-esteem, engendering feelings of discomfort. This issue is intensified for individuals residing in rural areas, where outdoor labor is routinely undertaken amidst challenging environmental conditions such as elevated temperatures, humidity, and exposure to the elements. The social ramifications of visible skin condition can also quickly mount due to typical dermatologist visits that can be financially straining and timely. Women who already face societal limitations may feel constrained by their skin condition and even withdraw from social interactions out of shame [11]. A skin condition may not always be concealed in the same way as other medical issues as it may be more visible, and this visibility can be a source of negative stigma, judgement, and stress.

Skin and psyche are inextricably linked to numerous skin illnesses that induce or lead to co-morbid psychological disorders, such as anxiety and depression, and may significantly impact a patients’ QOL [12]. Skin illnesses may have a negative influence on women’s mental health. According to studies [13–15], those with one or more illnesses or skin disorders are more likely to suffer from depression, anxiety, and stress. Those with chronic inflammatory skin conditions also report much greater levels of distress.

In some cases, this distress can be severe and long-lasting, leading to further mental health issues, including self-doubt, shame, lack of confidence, and social isolation [16]. The social stigma of suffering from a visible skin condition, multiple medical visits, and the need to apply daily unpleasant topical medications add to the disease burden [17]. The psychological difficulties caused by various skin disorders highlight the possible need for dermatological and psychiatric therapy [18]. Therefore, improving the efficacy of skin disease treatments necessitates providing psychological care and social support to individuals suffering from psychological issues [12]. In treating psychological sequelae, a patient’s social support system, including family and interpersonal relationships, coping abilities, and belief systems, play important roles [19]. In addition, improved awareness of the psychological comorbidity associated with skin illness and a biopsychosocial approach to care will eventually enhance patients’ lives [20]. The positive correlation between elevated levels of perceived social support and a reduced incidence of depressive symptoms underscores the pivotal role of social support in aiding women to navigate the emotional challenges associated with inflammatory skin conditions [21]. This connection emphasizes the significance of fostering supportive networks to contribute to women’s well-being in the face of such dermatological concerns. Social connections and relationships significantly determine people’s social and physical functioning and overall well-being. Social support have been associated with better mental and physical health [22].

### The study aims and questions

Based on the previous literature, the main goal of this study is to assess the QOL of women with vitiligo diseases within a traditional society. This research seeks to understand how these conditions impact day-to-day lives and to determine if particular treatments or interventions improve the well-being- of individuals with Vitiligo. Additionally, data gathered from this research may be used to develop improved treatment guidelines for women with Vitiligo or similar conditions and to inform healthcare providers on how best to support their patients. Using the Skindex-16 scale, the study assess the impact of vitiligo on the QOL of affected women. Ultimately, this research will provide meaningful insights into potential means of improving the lives of those dealing with skin diseases within traditional societies, such as those found in rural Libya.

The data collected were analyzed to develop a comprehensive understanding of the effects of vitiligo on women’s QOL. The results from this study provide valuable information to healthcare professionals, researchers, and policymakers to improve the quality of life for those living with skin diseases. As a result, this investigations key research question aimed to determine ‘*What is the impact of vitiligo on women’s quality of life?’*

## Methodology

### Data collection tools

The current study used the Skindex-16 scale to measure the impact of skin diseases on QOL. Developed by dermatologists, this self-administered questionnaire is designed to evaluate individuals’ perceptions and emotions concerning their skin problems and overall appearance [23].. The scale assesses 16-items, including symptoms, emotions, daily functioning, and treatment satisfaction. The reliability and validity of the scales have been consistently replicated and demonstrated in prior studies, particularly in research focusing on the QOL related to skin diseases. This includes various investigations such as clinical trials, epidemiologic studies, and surveys, as highlighted by Both et al. [24]. Furthermore, this scale has identified women in need of treatment and services and sheds light on the psychosocial effects of skin conditions. The Skindex-16 scale is a valuable tool for understanding the challenges associated with skin diseases and helping those affected.

The scale contains a dimension related to the disease (itching, pain, rough texture, recurrence of the disease), psychological factors (anxiety, frustration, embarrassment, anger, depression), as well as social counting (interaction, socialization, relationships, daily activity, and work performance). The Skindex-16 scale has also been used previously within Arab samples, to measure patients’ QOL and skin diseases [25, 26]. A pilot investigation was utilised using five participants to assess the main data collection tool, and modifications were made to paragraphs and phrases of the questions, based on the feedback of the observations.

### Sample

The research methodology adopts a case selection strategy focusing on individuals afflicted with dermatological disorders. However, recruiting patient participation posed considerable challenges due to the impediments imposed by their skin conditions, rendering their engagement in social activities notably intricate. The selection of the city of Ajdabiya was guided by a set of considerations stemming from its distinctive attributes. Notably, the city encompasses a robust traditional lifestyle, which the authors posit exerts significant influence on various health-related behaviors and attitudes. The researchers selected a dermatological clinic within the public health sector in Ajdabiya, overseen by the Libyan Ministry of Health. This clinic operates on weekdays, Sunday through Thursday, from 8 a.m. to 2 p.m. and is staffed by four female doctors who provide their services pro bono to patients. On average, the clinic attends to approximately thirty new female patients daily, with a subset comprising 3–6 individuals affected by leprosy or vitiligo. Upon securing necessary consents, one of the attending doctors facilitated patient engagement in the study. This involved the elucidation of the study’s scientific nature, emphasizing the absence of any information that could compromise patient identity, residence, or future well-being. Patients expressing consent were subsequently administered the questionnaire under the doctor’s supervision, creating an opportunity for additional discussion and addressing any concerns. However, the response rate was notably low, with a significant proportion declining participation, thereby diminishing the feasibility of accumulating a sufficiently sizable sample. The weekly average of questionnaire responses stood at approximately four instances. As delineated in the ensuing table, data collection extended over a duration of 20 weeks (Table 1).

**Table 1:** Data collection for a total of 20 weeks

Weak No	W1	W2	W3	W4	W5	W6	W7	W8	W9	W10	W11	W12	W13	W14	W15	W16	W17	W18	W19	W20	Total
weakly patient	42	38	35	37	33	31	40	42	37	44	27	33	39	33	31	36	29	30	32	38	669
Vitiligo and leprosy patients	4	2	5	6	2	4	6	7	5	8	5	3	4	3	2	3	4	5	5	4	83
Sample	2	2	3	5	2	3	6	4	5	6	3	2	4	1	2	2	3	4	3	3	65

Table 2 delineates the frequencies and corresponding percentages for each variable characterizing the sample.

**Table 2:** Sample characteristics (*n* = 65)

Characteristic	n	%
Age group		
Below 20	12	18.46
20–29	17	26.15
30–39	10	15.38
40–49	10	15.38
50+	16	24.62
Education level		
Non-university graduates	45	69.23
University degree graduates	20	30.77
Employment status		
Employed	52	80.00
Unemployed	13	20.00
Monthly income		
Low	40	61.54
Average	15	23.08
High	10	15.38
Relationship status		
In a relationship	35	53.85
Not in a relationship	30	46.15
Number of family member with vitiligo.		
One member	41	63.08
Two or more	24	36.92
How long have you had vitiligo symptoms?		
One year or less	13	80.00
More than one year	52	20.00

### Psychometric properties

Using Cronbach’s alpha coefficient, 0.7 is considered to be the minimum acceptable reliability coefficient for the developed questionnaire and individual scales. Cronbach’s alpha for the entire instrument was found to be 0.877, indicating an extremely high level of internal consistency. Furthermore, as shown in Table 3, Cronbach’s Alpha was 0.903, 0.912, and 0.849 for the scales denoting symptoms, emotions, and functioning, respectively. The validity of the instrument was evaluated using a confirmatory factor analysis (CFA) as well as a goodness of fit analysis of the supposed three-domain structure. According to Table 3, all items in the model have factor loadings between 0.621 and 0.967. Given the small sample size of this study, a minimum factor loading of 0.6 can be considered acceptable [27, 28]. Based on the recommendations made by Henseler et al. [29], the researchers calculated the average variance extracted (AVE) to be greater than or equal to 0.50. According to Table 3, the AVE ranges from 0.624 to 0.903. As a final assessment, the Standardized Root Mean Squared Residual (SRMR) was calculated and expected to be less than 0.08 [30]. A SRMR of 0.076 met the cut-off criteria in the current study.

**Table 3:** Measurement statistics for instrument validity and reliability

Construct	Item	Loading	Cronbach’s Alpha	AVE
Symptoms	S1	0.912	0.964	0.903
	S2	0.967		
	S3	0.961		
	S4	0.960		
Emotions	E1	0.663	0.912	0.653
	E2	0.621		
	E3	0.642		
	E4	0.917		
	E5	0.879		
	E6	0.918		
	E7	0.936		
Functional	F1	0.721	0.849	0.624
	F2	0.804		
	F3	0.871		
	F4	0.729		
	F5	0.816		

### Data analysis

The analyses were conducted with SPSS version-29 and Smart-PLS version 4. A mean and standard deviation, as well as a frequency, were computed to represent the data. Additionally, since the sample size was small, the non-parametric test “Mann-Whitney U” was used to compare the Skindex-16 scores of the patients based on their individual characteristics. In order to analyze the correlation between variables, the Spearman correlation test was applied. *p*-values less than 0.05 were used to determine the significance of the results.

## Results

Table 4 below shows the mean (*M*), standard deviation (*SD*), for the 16-items included in the survey. A range of 1.80 to 4.015 was observed for the item means. According to the mean values, item E2 had the highest mean value of 4.015 (*SD* = 0.625). Conversely, the lowest ranked item was item S2 with a mean value of 1.80 (*SD* = 0.592).

**Table 4:** Mean, standard deviation, for survey items (*n* = 65)

Items	During the past week, how much have you been bothered by:	*M*	*SD*
*Symptoms*	*1.839*	*0.611*	
S1	Your skin condition itches	1.862	0.704
S2	Your skin condition burns or stings	1.800	0.592
S3	Your skin condition hurts	1.804	0.618
S4	Your skin condition irritates	1.892	0.664
*Emotions*	*3.846*	*0.623*	
E1	The persistence/reoccurrence of your skin condition	3.523	1.032
E2	Worry about your skin condition	4.015	0.625
E3	The appearance of your skin condition	4.000	0.468
E4	Frustration about your skin condition	3.877	0.696
E5	Embarrassment about your skin condition	3.969	0.612
E6	Being annoyed about your skin condition	3.754	1.001
E7	Feeling depressed about your skin condition	3.785	0.927
*Functional*	*2.739*	*0.828*	
F1	The effects of your skin condition on your interactions with others	3.354	1.124
F2	The effects of your skin condition on your desire to be with people	3.200	1.148
F3	Your skin condition is making it hard to show affection	2.692	1.060
F4	The effects of your skin condition on your daily activities	2.123	0.875
F5	Your skin condition makes it hard for you to work	2.323	1.017
*Quality of life*		*2.998*	*0.505*

### Mann-Whitney U test

The Mann-Whitney U test was performed to compare the distributions of different groups in terms of symptoms, emotions, functional, and quality of life scores, as shown in Table 5.

**Table 5:** Mann-Whitney U tests for symptoms, emotions, functional, and quality of life score by different characteristics

Dependent variable	Independent variable	Group	Mean Rank	U	z	p
Symptoms	Education level	Non-university graduates	32.17	412.50	0.628	0.530
		University degree graduates	34.88			
	Employment status	Employed	32.47	310.50	0.531	0.595
		Unemployed	35.12			
	Relationship status	In a relationship	32.23	498.00	0.419	0.675
		Not in a relationship	33.90			
	Number of family members with vitiligo or leprosy.	One	35.73	380.00	1.794	0.073
		Two or more	28.33			
	How long have you had the disease symptoms?	1 year or less	28.96	285.50	1.015	0.310
		More than 1 year	34.01			
Emotions	Education level	Non-university graduates	33.86	411.50	0.563	0.574
		University degree graduates	31.08			
	Employment status	Employed	31.34	251.50	1.458	0.145
		Unemployed	39.65			
	Relationship status	In a relationship	41.30	234.50	3.930	0.000
		Not in a relationship	23.32			
	Number of family members with vitiligo and leprosy.	One	31.57	433.50	0.817	0.414
		Two or more	35.44			
	How long have you had the disease symptoms?	1 year or less	32.27	328.50	0.160	0.837
		More than 1 year	33.18			
Functional	Education level	Non-university graduates	33.98	406.00	0.630	0.529
		University degree graduates	30.80			
	Employment status	Employed	29.75	169.00	2.792	0.005
		Unemployed	46.00			
	Relationship status	In a relationship	36.79	392.50	1.756	0.079
		Not in a relationship	28.58			
	Number of family members with vitiligo or leprosy.	One	29.96	367.50	1.705	0.088
		Two or more	38.19			
	How long have you had the disease symptoms?	1 year or less	36.58	291.50	0.768	0.442
		More than 1 year	32.11			
Quality of life	Education level	Non-university graduates	33.80	414.00	0.513	0.608
		University degree graduates	31.20			
	Employment status	Employed	30.33	199.00	2.283	0.022
		Unemployed	43.69			
	Relationship status	In a relationship	38.70	325.50	2.630	0.009
		Not in a relationship	26.35			
	Number of family members with vitiligo or leprosy.	One	30.68	397.00	1.293	0.196
		Two or more	36.96			
	How long have you had the disease symptoms?	1 year or less	33.35	333.50	0.074	0.941
		More than 1 year	32.91			

Table 5 indicates that there is a statistically significant difference between women in a relationship and others in terms of emotional scores, with the first group having a higher mean rank (*U* = 234.5, z = 3.930, *p* = .001). According to the Mann-Whitney U test, there was a significant difference between the two groups, suggesting that relationship status affects the emotional score of women. Further, employed women and unemployed women have statistically significant differences in functional scores, with the employed women having a lower mean rank than the second (U = 169, z = 2.792, *p* = 0.005). As indicated by the Mann-Whitney U test, there was a significant difference between the two groups, suggesting that women’s employment status has a significant impact on their functional scores. Based on the QOL (overall) score, it was found that employed women had a significantly lower mean rank compared to unemployed women (U = 199, z = 2.283, *p* = 0.022). Additionally, there is a statistically significant difference between women in a relation and women not in a relationship (U = 325.5, z = 2.630, *p* = 0.009), with the first group having a higher mean rank than the second group.

### Spearman correlation

Spearman correlation was performed to examine the association between different survey domains and several demographic variables such as age group and monthly income. Results are shown in Table 6.

**Table 6:** Spearman’s rank correlation coefficient

Variable	Symptoms	Emotions	Functional	Quality of life
Age	0.192 (0.125)	−0.094 (0.456)	− 0.033 (0.796)	− 0.016 (0.901)
Monthly Income	0.147 (0.242)	− 0.256 (*0.040*)	− 0.252 (*0.043*)	− 0.276 (*0.026*)

Table 6 shows a significant, negative correlation between monthly income and the emotion score, functional score, and the QOL score.

## Discussion

While vitiligo has been prevalent in Libya for an extended period, the subject has only recently garnered attention within the realm of health science research [31, 32] that focuses on issues regarding treatment and drug efficacy. The current study is the first thorough investigation that has assessed the impact of vitiligo disease on women’s QOL in Libya. The main objective of this study was to establish how vitiligo affects women’s QOL in a traditional community. Our results showed that age was important in both the severity of illness and the QOL for patients, with younger women exhibiting higher anxiety levels than older women due to concerns regarding the here and now and future. According to Amer & Gao [33], patients’ QOL, emotions, and future concerns are profoundly affected by demographic characteristics such as age and the affected body area. The current study’s results also indicate a close relationship between women’s infection with vitiligo disease and exclusion by society concerning marriage and partner selection. Studies [34, 35] indicate that this is also related to the lack of marriage opportunities among women with skin diseases in traditional societies, as the relationship with the opposite sex and skin diseases negatively affect marriage. Vitiligo can be characterized as a condition with pronounced social isolation, stemming from both its conspicuous physical symptoms and the associated stigma arising from alterations in skin appearance [21].

The present study affirms that women grappling with this condition experience social pressures. Consequently, females find themselves in a state of social isolation within both their immediate and broader social environments. This is in line with the findings of Pärna et al. [36], who found that visible cutaneous symptoms and the need for treatment were associated with reduced participation in social activities. As such, women with skin conditions may experience lower self-esteem and more stress in their daily interactions with friends and family. Women with skin diseases face significant social stigma in these communities, which can hinder their ability to find romantic partners and even prevent marriage in some cases [37, 38]. The study found that the disease impacts the patient’s emotions, leaving them weak and unable to cope with their surroundings. The patient’s QOL and capacity for social interaction suffer due to losing confidence, which is tied to the patient’s emotional state. Patients in the current study felt the emotional toll of living with this disease. According to research published in 2008 by Magin et al., patients with skin diseases may be more open to ridicule, which can have serious psychological consequences.

The current study demonstrated that women’s employment status was another factor of interest in influencing their emotions towards their condition. The results showed that patients with employment were more likely to cope positively with their illness and experience fewer negative emotions than those without employment. This suggests that the professional status of female workers positively affects the general condition of women with vitiligo, even though there is a fear of bullying or discrimination in the workplace. Yew et al. [39] found that women who were unable to find employment due to a skin disease were more likely to be depressed than those who were employed despite having the same condition.

Although the economic status did not appear to influence the patient’s emotional well-being, it significantly affected their overall QOL. Pahwa et al. [40] indicated that there is a relationship between the economic status of women with vitiligo and the QOL due to the financial burdens caused by the disease. Women with high incomes do not find it difficult to receive treatment, this indicates discrepancies in financial resources within the health system and the allocation of health services [41]. This phenomenon has adverse implications, particularly for women belonging to lower socio-economic strata. This disparity is frequently conspicuous in traditional societies where female poverty is prevalent, resulting in a diminished QOL when confronted with inflammatory skin diseases. The current study shows that women with vitiligo face a reduced QOL in a society dominated by a traditional gender pattern.

### Implication for social work practice and research

The current study’s findings highlight the severe consequences of skin diseases for women residing within a North African country and the significance of providing prompt, effective care. The heightened emphasis on privacy and secrecy among women throughout the study can be attributed to their coping mechanisms in the face of chronic diseases. Emotional distress, manifested through symptoms such as insomnia and general anxiety, has been demonstrated to impede a woman’s capacity to engage fully in various aspects of life [36]. Participants with skin conditions more commonly reported depression, social withdrawal, loneliness, and a diminished QOL. Patients who were unemployed, single, or elderly were more likely to show signs of depression. Mitigating the burden of skin diseases necessitates heightened consideration of the psychological dimensions of care. Notably, early psychosocial interventions and vigilant monitoring for mood changes are pivotal in this context [39]. By integrating the specific needs of female patients and acknowledging their health preservation strategies, a novel model can be formulated to guide practitioners in delivering more effective and comprehensive care. In this setting, and considering the social, cultural, and economic circumstances of Libyan society as a North African society, we observe a de facto lack of the social worker’s role in the medical sector. Therefore, these women cannot access acceptable forms of social support. Therefore, this may lead to patients developing their own strategies to compensate for a lack of quality healthcare system that focuses primarily on medical aspect. To provide the best possible care for these women and protect their right to a high QOL, the medical system must adopt a model that combines health intervention with official social support intervention [42, 43]. Integrating feminist theories into social work involves acknowledging and confronting systemic inequalities that impact marginalized groups, as outlined by Fisher-Borne et al. [44]. This extends to encompass women with skin diseases in North Africa, necessitating a comprehensive approach to address their unique challenges within the broader framework of social justice. It also involves promoting a more holistic and comprehensive approach to care, one that considers not only the medical aspect but also the social and psychological dimensions. This can ultimately lead to a more inclusive and supportive environment for individuals facing skin diseases, where they can access both quality medical care and appropriate forms of social support.

### Limitations

This study focuses specifically on a case study from North Africa, which serves as an example for other similar contexts. A limitation of this research is its exclusive focus on the impact of these issues on QOL, neglecting other crucial dimensions such as social stigma, coping mechanisms, healthcare access, psychosocial support, cultural influences, and economic implications. This study does not include other external factors, such as the economic costs and impact on social relationships. It is crucial to acknowledge that these additional factors contribute to the holistic understanding of the subject, emphasizing the need for further research to unravel their specific roles and contributions. Due to the unique characteristics of this topic, collecting a representative sample has proven to be challenging, with a response rate of approximately 9.7%, suggesting that other sampling methods should be utilised in future investigations. It is also important to recognize that these results may not be generalized to all traditional societies, as they depend heavily on a specific cultural context. Ultimately, this research provides valuable insight into how vitiligo disease can affect the QOL among women within traditional societies, allowing for a better understanding of the issue. However, further research is necessary to understand these conditions’ more comprehensively.

## Conclusion

The results of this study on women with vitiligo in the North African region indicate that skin diseases can significantly impact the QOL. Traditional social norms and values play an important role in how these women are perceived by their society; thus, they risk feeling isolated and discriminated against. These feelings can hinder their ability to live a fulfilling and healthy life. Therefore, it is necessary to address the social stigma of skin diseases in traditional society so that women living with such conditions can access appropriate care and resources. This will ultimately improve their overall QOL.

In conclusion, this study highlights the need for increased awareness and greater support for women with skin conditions to help them lead healthy and fulfilling lives. There is a need to support women with vitiligo and other inflammatory skin diseases by introducing a social work unit within health centers in Libya, which should focus on adopting feminist approach given the marginalization faced by women with vitiligo. This will enable formal social support for women in Libya and enhance their QOL.

## Data Availability

Due to privacy restrictions, the data used in this study are available upon request from the authors.
